# Why Does Cronobacter sakazakii Survive for a Long Time in Dry Environments? Contribution of the Glass Transition of Dried Bacterial Cells

**DOI:** 10.1128/spectrum.01384-21

**Published:** 2021-12-15

**Authors:** Kyeongmin Lee, Kento Koyama, Kiyoshi Kawai, Shigenobu Koseki

**Affiliations:** a Graduate School of Agricultural Science, Hokkaido University, Sapporo, Hokkaido, Japan; b Graduate School of Integrated Sciences for Life, Hiroshima University, Higashi-Hiroshima, Hiroshima, Japan; Health Canada

**Keywords:** desiccation tolerance, freeze-dry, glass transition temperature, storage temperature, water activity

## Abstract

To investigate the mechanism of adaptation of Cronobacter sakazakii to desiccation stress, the present study focused on the glass transition phenomenon of dried bacterial cells, using a thermomechanical technique. The mechanical glass transition temperature (*T_g_*) of dried C. sakazakii cells *per se*, prepared by different drying methods (air drying and freeze-drying) and with different water activity (a_w_) levels (0.43, 0.57, 0.75, and 0.87), were determined. In addition, we investigated the survival of two strains of C. sakazakii (JCM 1233 and JCM 2127) prepared by different drying methods under different storage temperatures (4, 25, and 42°C) and a_w_ conditions (0.43 and 0.87). While the *T_g_* of the air-dried C. sakazakii cells increased as the a_w_ decreased, the freeze-dried C. sakazakii cells showed an unclear a_w_ dependency of the *T_g_*. Air-dried C. sakazakii cells showed a higher *T_g_* than freeze-dried C. sakazakii cells at an a_w_ of <0.57. Freeze-dried C. sakazakii cells were more rapidly inactivated than air-dried cells regardless of the difference in a_w_ and temperature. The difference between the *T_g_* and storage temperature was used as an index that took into consideration the differences in the drying methods and a_w_ levels. As the difference between the *T_g_* and storage temperature increased to >20°C, the dried C. sakazakii cells survived stably regardless of the drying method. In contrast, when the difference between the *T_g_* and storage temperature was reduced to <10°C, the viable cell numbers in dried C. sakazakii cells were quickly decreased. Thus, the *T_g_* is a key factor affecting the desiccation tolerance of C. sakazakii.

**IMPORTANCE** The mechanical glass transition temperature (*T_g_*) of dried Cronobacter sakazakii cells varied depending on differences in drying methods and water activity (a_w_) levels. Because the *T_g_* of the dried bacterial cells varied depending on the drying method and a_w_, the *T_g_* will play an important role as an operational factor in the optimization of dry food processing for controlling microbial contamination in the future. Furthermore, the differences between the *T_g_* and storage temperature were introduced as an integrated index for survival of bacterial cells under a desiccation environment that took into consideration the differences in the drying methods and a_w_ levels. As the difference between the *T_g_* and storage temperature decreased to <10°C, the dried C. sakazakii cells were inactivated quickly, regardless of the drying methods. The relationship between *T_g_* and storage temperature will contribute to understanding the desiccation tolerance of bacterial cells.

## INTRODUCTION

Outbreaks of foodborne diseases have recently and occasionally been related to low-moisture foods. Salmonella spp., Bacillus cereus, Clostridium spp., Escherichia coli O157:H7, Staphylococcus aureus, and Cronobacter sakazakii are the main foodborne pathogens responsible for foodborne disease outbreaks related to dried foods (1–7). There are some reports showing that most of these bacteria can survive for long periods, such as more than a year, under desiccation (1–3). For example, C. sakazakii, Salmonella spp., and E. coli O157:H7 can persist for more than 12 months in powdered infant formula (4, 5). Although storage temperature and water activity (a_w_) have been thought to be critical factors for the long-term survival of bacteria in dry environments, the governing environmental or intrinsic factors underpinning the survival of bacteria have not been elucidated thus far (3, 6).

Identification of the critical and governing environmental or intrinsic factors associated with the desiccation tolerance of bacteria is expected to contribute significantly to the control of foodborne pathogens contaminating and surviving in dry foods for long periods (6). The governing factors can then be applied for optimization of dry food processing and/or distribution. Although there have been some reports on evidence for factors that are protective to C. sakazakii cells under desiccation, such as trehalose accumulation (8) and the presence of capsules (9–11), the mechanism underpinning bacterial survival in dry environments could be partly elucidated by the identification of previously unknown critical factors. Thus, the present study aimed to identify the factors governing the long-term survival of pathogenic bacteria in dry environments.

Storage temperature and water activity (a_w_) cannot sufficiently explain bacterial desiccation tolerance, which has long been studied by many researchers (6, 7, 11–15). These factors alone could not sufficiently explain properties like the fluidity of the bacterial cytoplasm. Thus, we introduced the concepts of glassy state and glass transition phenomenon as a third evaluation axis related to cryptobiosis (16). The cryptobiosis state has been observed in some organisms, such as tardigrades and African chironomids (17, 18). Cryptobiosis is a metabolic state that confers a high survival rate on organisms by transforming them under harsh environments, such as in deserts. For the efficient preparation of probiotics that show some health effects, such as lactic acid bacteria, the glass transition phenomenon and control of the glass transition temperature (*T_g_*) of freeze-dried lactic acid bacteria containing protectants have been considered additional and/or alternative control factors to temperature and a_w_ (19). Accordingly, we hypothesized that the glass transition phenomenon might explain the survival of pathogenic bacteria under desiccation. In addition, we further focused on the relevance of the effect of the drying method on the survival and/or glass transition of bacterial cells. Because drying and glass transition are both highly related to the movement of water molecules in the material, the drying method might also have an impact on the desiccation tolerance of bacteria.

In the present study, we focused on C. sakazakii, which has long been a concern with regard to the safety of powdered infant formula, as a representative desiccation-tolerant bacterium (20–27). The objectives of this study were to determine and compare the mechanical *T_g_* of dried C. sakazakii cells prepared by different drying methods and with different a_w_ levels, using a thermomechanical technique. In addition, the survival of C. sakazakii cells during storage under different a_w_ levels and temperatures was investigated, and the relationship between storage temperature and *T_g_* in the survival of C. sakazakii is discussed.

## RESULTS

### Glass transition temperature of C. sakazakii cells.

**FIG 1:**
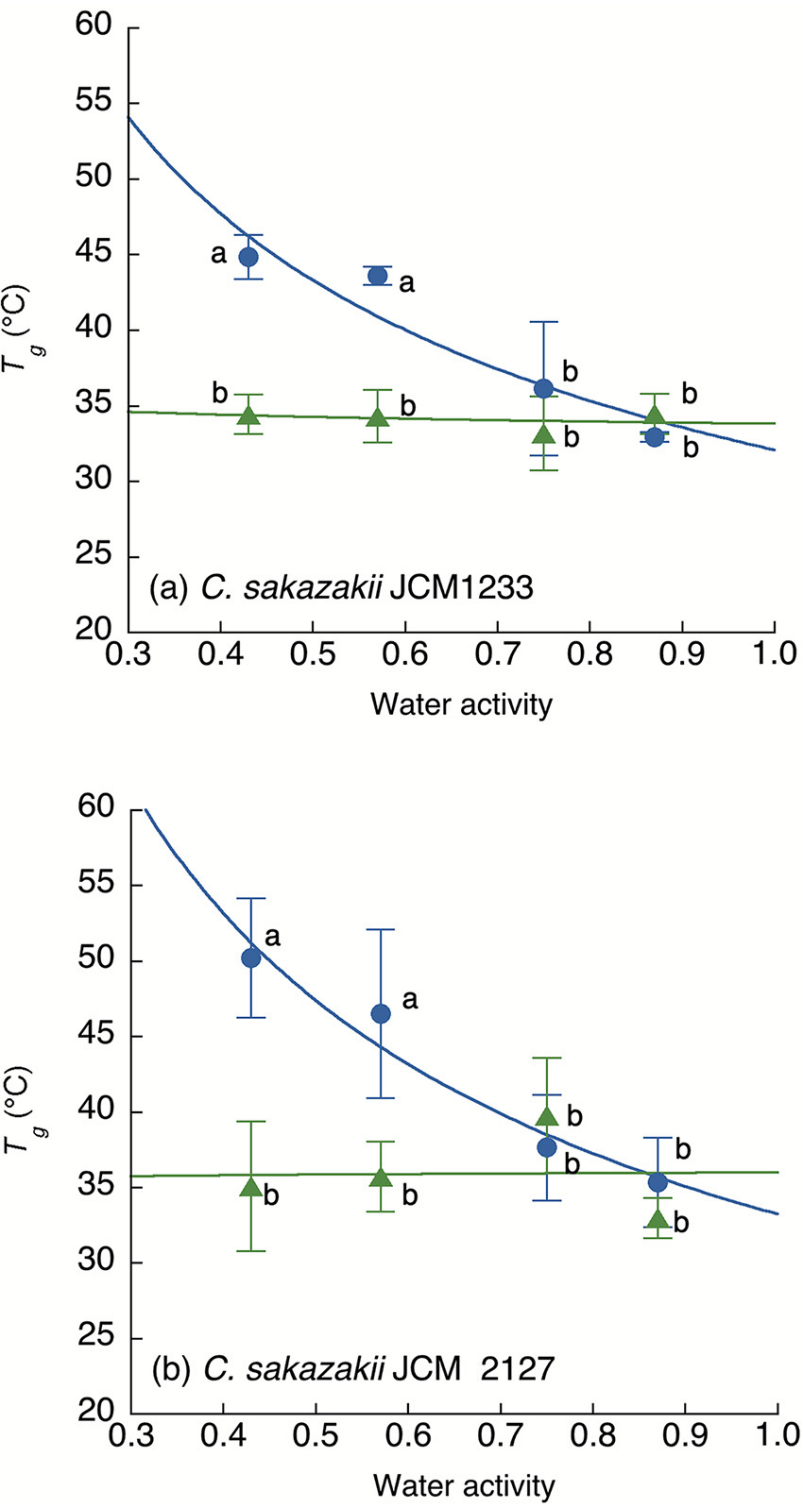
Relationship between the water activity (a_w_) and observed mechanical glass transition temperature (*T_g_*) of air-dried (●) and freeze-dried (▴) Cronobacter sakazakii strains JCM 1233 (a) and JCM 2127 (b). The results are expressed as the mean values ± standard deviations from three independent experiments. Values with different letters at the same a_w_ between the air-drying and freeze-drying methods were significantly different (*P < *0.05).

The relationship between the a_w_ and *T_g_* of C. sakazakii cells differed significantly between the drying methods, as shown by the results in Fig. 1. While air-dried C. sakazakii cells exhibited a decrease in *T_g_* with increasing a_w_, freeze-dried C. sakazakii cells exhibited an unclear *a*_w_ dependency of the *T_g_*. The *T_g_* values were significantly lower than those of air-dried samples at lower a_w_ values, such as a_w_ 0.43 (*P = *0.00012 and 0.00031 for strains JCM 1233 and JCM 2127, respectively) and a_w_ 0.57 (*P = *0.00045 and 0.00021 for strains JCM 1233 and JCM 2127, respectively). The *T_g_* of air-dried C. sakazakii cells at an a_w_ of <0.57 was approximately 10 to 15°C higher than that of freeze-dried C. sakazakii cells. No significant difference in *T_g_* between the two drying methods was observed under high a_w_ levels (>0.75; *P > *0.05 for both strains). These trends were observed regardless of differences between the C. sakazakii strains, and there was no significant difference in the *T_g_* values at the same a_w_ between the two strains.

### Survival kinetics of C. sakazakii cells during storage.

**FIG 2:**
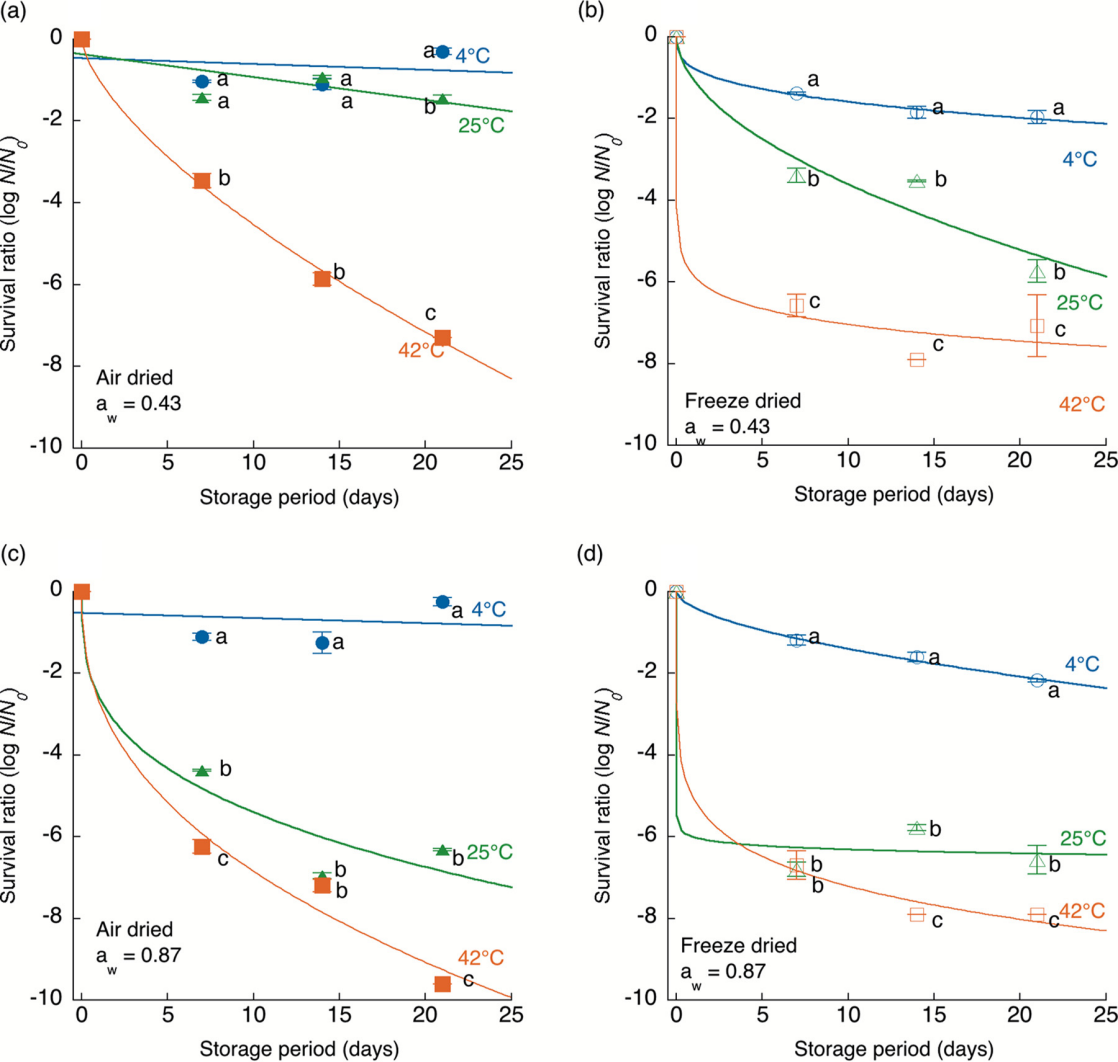
Survival kinetics of air-dried (a_w_ 0.43 [a] and 0.87 [c]) Cronobacter sakazakii (JCM 1233) cells during storage at 4°C (●), 25°C (▴), and 42°C (■) and freeze-dried (a_w_ 0.43 [b] and 0.87 [d]) C. sakazakii cells during storage at 4°C (○), 25°C (△), and 42°C (□). The initial cell numbers (*N*_0_) were 10^8^ to 10^9^ CFU/g. The results are expressed as the mean values ± standard deviations from three independent experiments. The curves were described by the Weibull model. Values with different letters at the same sampling time among different conditions are significantly different (*P < *0.05).

**FIG 3:**
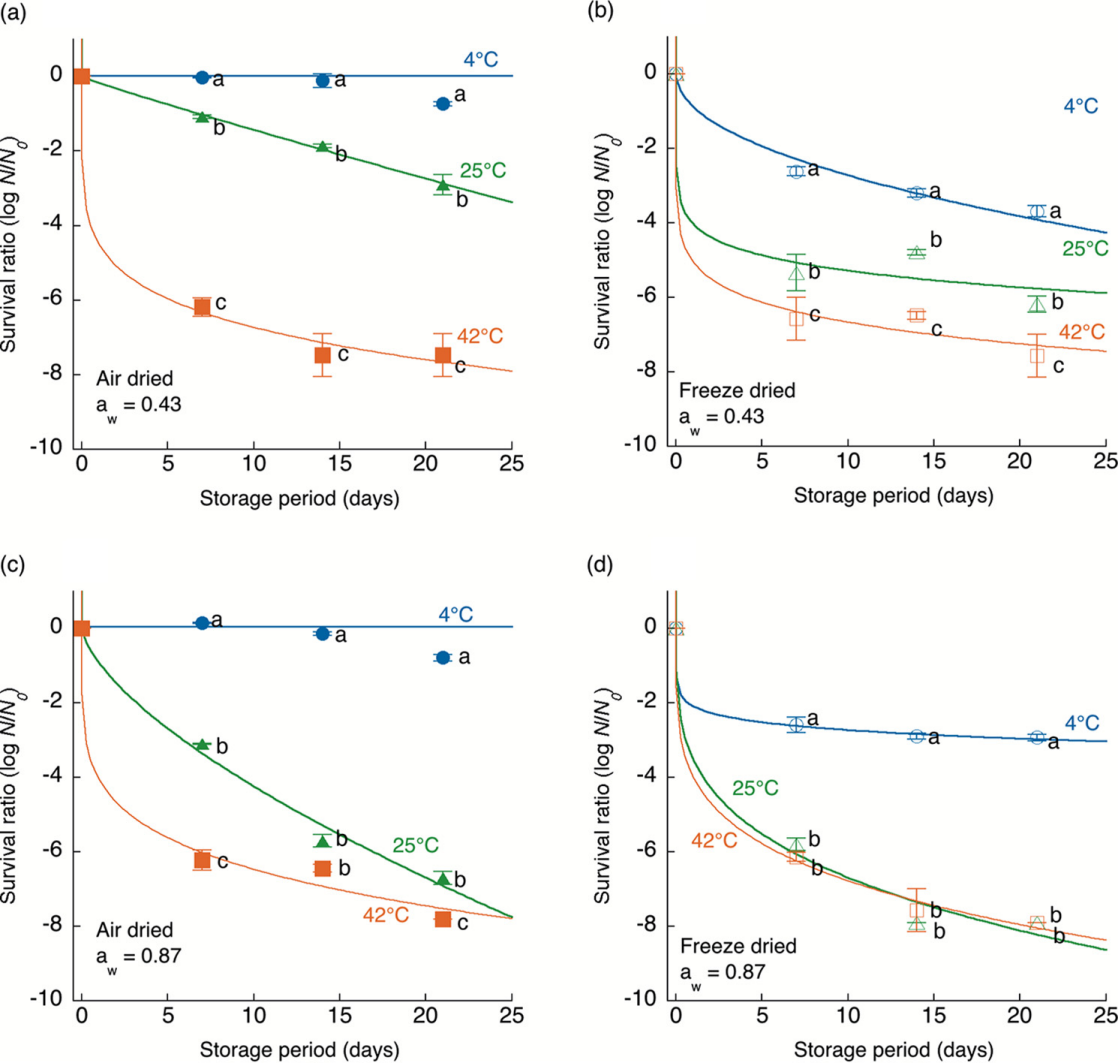
Survival kinetics of air-dried (a_w_ 0.43 [a] and 0.87 [c]) Cronobacter sakazakii (JCM 2127) cells during storage at 4°C (●), 25°C (▴), and 42°C (■) and freeze-dried (a_w_ 0.43 [b] and 0.87 [d]) C. sakazakii cells during storage at 4°C (○), 25°C (△), and 42°C (□). The initial cell numbers (*N*_0_) were 10^8^ to 10^9^ CFU/g. The results are expressed as the means ± standard deviations of three independent experiments. The curves were described by the Weibull model. Values with different letters at the same sampling time among different conditions were significantly different (*P < *0.05).

Storage temperature greatly influenced the survival kinetics of dried C. sakazakii cells regardless of differences in drying methods (air or freeze-dried), a_w_ levels (0.43 or 0.87), and strains (JCM 1233 and 2127), as shown by the results in Fig. 2 and 3. The higher the storage temperature applied was, the faster the death rate. The fitted death rate parameters (*b*) of the survival kinetics clearly illustrated the trend of faster death at higher storage temperatures (Table 1). The drying method also affected the survival kinetics of dried C. sakazakii cells. The surviving cell numbers of freeze-dried C. sakazakii decreased more rapidly than those of air-dried C. sakazakii regardless of the storage temperature, a_w_, and strain. In particular, marked differences in the survival kinetics of C. sakazakii cells stored at 25°C and a_w_ 0.43 were observed between the drying methods for both strains. The survival kinetics of C. sakazakii cells stored at a_w_ 0.87 did not exhibit a large difference between the drying methods at any of the tested temperatures compared with the differences observed under storage at a_w_ 0.43. The influence of storage temperature and drying method was pronounced at lower a_w_ levels, such as a_w_ 0.43. In summary, the results demonstrated that the higher the a_w_ and storage temperature were, the faster bacterial death occurred, regardless of the difference in the drying methods.

**TABLE 1:** Fitted Weibull parameters of survival kinetics of C. sakazakii cells dried by different methods during storage at different temperatures and water activities

Temp	Drying method	a_w_	Value for indicated parameter in strain^*a*^:					
			JCM 1233			JCM 2127		
			*b*	*n*	*R* ^2^	*b*	*n*	*R* ^2^
4°C	Air dry	0.43	0.01	0.99	0.99	0.02	2.28e−3	0.99
		0.87	0.01	0.99	0.99	0.05	5.38e−4	0.97
	Freeze-dry	0.43	0.76	0.32	0.98	0.88	0.49	0.96
		0.87	0.38	0.57	0.99	2.08	0.12	0.97

25°C	Air dry	0.43	0.05	0.99	0.97	0.16	0.94	0.95
		0.87	2.58	0.32	0.98	0.93	0.66	0.96
	Freeze-dry	0.43	1.06	0.53	0.99	4.03	0.12	0.97
		0.87	6	0.02	0.97	3.52	0.28	0.95

42°C	Air dry	0.43	0.99	0.66	0.96	4.49	0.18	0.97
		0.87	2.68	0.41	0.97	4.05	0.20	0.95
	Freeze-dry	0.43	5.84	0.08	0.98	5.04	0.12	0.96
		0.87	5.06	0.15	0.97	3.98	0.23	0.97

a *b*, survival rate; *n*, survival kinetics curvature.

### Effect of the relationship between storage temperature and *T_g_* on the survival of C. sakazakii cells.

**FIG 4:**
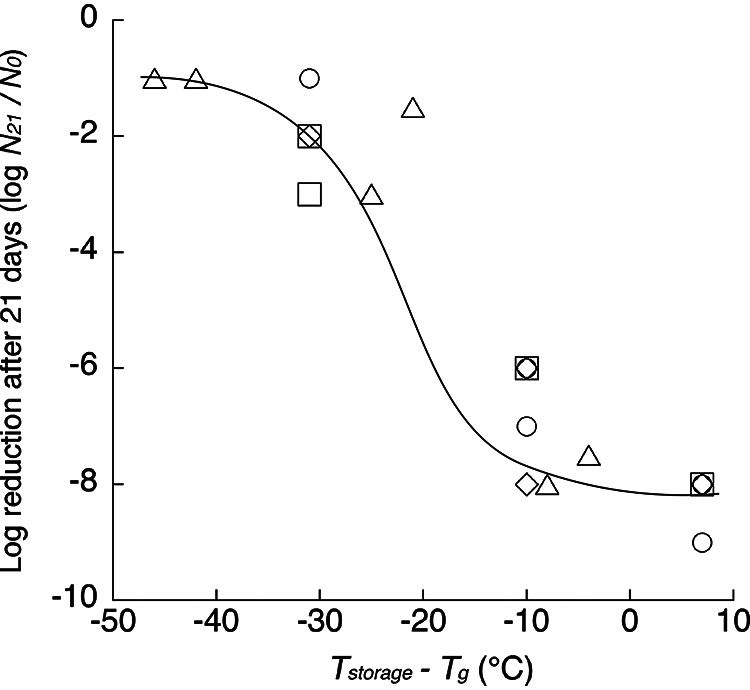
Effect of the difference in mechanical glass transition temperature (*T_g_*) and storage temperature (*T*_storage_) on the log reduction in C. sakazakii (JCM 1233 and JCM 2127) cells prepared by air drying and freeze-drying after 21 days of storage and at an adjusted a_w_ of 0.43 or 0.87. *N*_0_ and *N*_21_ indicate the initial viable cell number and the viable cell number after 21 days of storage, respectively. ○, air dried, with a_w_ of 0.43; △, air dried, with a_w_ of 0.87; □, freeze-dried, with a_w_ of 0.43; ◊, freeze-dried, with a_w_ of 0.87.

We demonstrated the effect of the relationship between storage temperature and *T_g_* on the survival (log-cycle reduction after 21 days of storage) of dried C. sakazakii cells (Fig. 4). As the difference between *T_g_* and storage temperature increased to >20°C, slight reductions in viable cell numbers (1- to 2-log-cycle reductions) were observed regardless of differences in the drying methods and a_w_ levels. In contrast, when the difference between *T_g_* and storage temperature decreased to <10°C, the viable cell numbers of C. sakazakii were markedly decreased (8- to 9-log-cycle reductions) regardless of differences in the drying methods and a_w_ levels. The decreases in the viable cell numbers of C. sakazakii were dependent only on the relationship between *T_g_* and storage temperature and were independent of the drying method and a_w_ level. The results illustrated in Fig. 4 clearly indicate that the difference between *T_g_* and storage temperature plays a key role in explaining the survival of dried C. sakazakii cells during storage at different temperatures and under different a_w_ conditions, regardless of the drying methods.

## DISCUSSION

Differences in the drying methods used for C. sakazakii cells resulted in differences in the *T_g_* (Fig. 1) and survival kinetics (Fig. 2 and 3). The differences in the *T_g_*, as shown by the results in Fig. 1, could be attributed to the drying mechanism. The present study used air drying and freeze-drying as representative methods for dry food production. Since both methods are widely used for the production of various forms of dry food, the effect of those drying methods on the survival of dried bacterial cells would be applicable to various kinds of dry food. While the freeze-drying method removes solid water from bacterial cells by sublimination and results in a porous state, the air-drying method gradually removes water by evaporation. The desiccation speed, process, and phase transition of water might influence the amorphous components, and slow desiccation by air drying might result in a higher *T_g_*. Although the spray-drying procedure is also used in powder production, especially in the production of powdered infant formula, rapid evaporation by spray drying might have results similar to those of freeze-drying. The effect of spray drying on the survival of dried bacterial cells should also be investigated in the future. Furthermore, the effect of the desiccation rate on the glass transition needs to be investigated to further our understanding of the mechanism underlying the differences in *T_g_* among different drying methods.

The survival kinetics of dried C. sakazakii cells in the present study were overall consistent with those from previous studies (5, 22–24). As the storage temperature and a_w_ increased, the viable cell numbers of C. sakazakii decreased more rapidly. In contrast, a difference in the survival kinetics between air-dried and freeze-dried samples was observed, even at the same storage temperature and a_w_. Freeze-dried C. sakazakii cells were more rapidly inactivated than air-dried C. sakazakii cells (Fig. 2 and 3). A previous study showed that fluidized bed drying, an air-drying method, supports better survival of Lactobacillus paracasei than freeze-drying because it causes reduced absorption of water, which is attributed to the lower porosity and larger agglomerates (28). In contrast, fluidized bed drying significantly reduced the survival rates of Enterococcus faecium and Lactobacillus plantarum compared to freeze-drying (29). In addition, some viable bacteria were detected even after freeze-drying, and their survival rates varied from 8.0 to 96.7% over 20 years (16, 30). Since the differences in bacterial survival between the drying methods have not been sufficiently investigated in detail thus far, there has been a paucity of research on the effect of the drying method on the viability of foodborne pathogens.

The present study demonstrated the possibility of using *T_g_* as an alternative and integrated index for evaluating multiple effects of temperature, a_w_, and drying method on the survival of dried pathogenic bacterial cells. The importance of the difference between the environmental temperature and *T_g_* rather than *T_g_* itself was suggested as a critical factor affecting the physical state of amorphous polymers (19, 31). The so-called Williams-Landel-Ferry (WLF) equation is one example of such a relationship (31). Measurement of the viscosity of simple mono- and disaccharides revealed that the viscosity decreased by approximately 4 to 6 orders of magnitude when the temperature increased to 20°C above the *T_g_* (32–34). The stability of freeze-dried Lactobacillus paracasei subsp. paracasei cells was demonstrated at storage temperatures that were more than 20°C above the *T_g_* (19). The effect of the difference between temperature and *T_g_* on the survival of C. sakazakii cells was demonstrated in the present study, as well as in previous studies, as mentioned above. When the difference between the *T_g_* of the dried bacterial cells and storage temperature is sufficiently large, such as >20°C, the dried bacterial cells survive stably for a long period. In contrast, when the difference between the *T_g_* of the dried bacterial cells and storage temperature becomes small, such as <10°C, that is, if the *T_g_* is close to the storage temperature, the dried bacterial cells are inactivated quickly. The difference between the *T_g_* and storage temperature indicates whether the bacterial cells are in a stable glassy state or active rubbery state. Thus, the *T_g_* indicates whether the bacterial cells are tolerant of desiccation.

The protective effects of different kinds of sugars on the stability of bacterial survival were reported in previous studies (35, 36). The studies on the relationship between *T_g_* and the stability of bacterial cells so far included different kinds of sugars with dried bacterial cells. Those studies might not evaluate the net response of bacterial cells to desiccation, because of the protective effect of added sugars. On the other hand, the present study demonstrated the response of dried bacterial cells themselves. The relationship between the *T_g_* and a_w_ of C. sakazakii cells demonstrated in the present study would appropriately reflect the net physical response of bacterial cells to desiccation stress. This difference from the previous studies would contribute to further understanding of the mechanism of acquisition of desiccation tolerance of bacterial cells.

Because the *T_g_* of the dried bacterial cells varied depending on the drying method and a_w_, the *T_g_* will play an important role as an operational factor in the optimization of dry food processing for controlling microbial contamination in the future. Further investigation of the *T_g_* of dried microbial cells for other bacterial species and strains will be needed for generalization of the glass transition phenomenon in microorganisms. In addition, the glass transition of microbial cells in dry food environments should be examined. Furthermore, the mechanism underlying changes in the glassy state and the effect of drying speed on the glass transition and *T_g_* should be investigated. Nevertheless, the present study illustrated that the difference between the *T_g_* and storage temperature could be a useful index for prediction of the survival kinetics of dried bacterial cells during storage.

## MATERIALS AND METHODS

### Preparation of dried bacterial cells.

C. sakazakii strains (JCM 1233 and JCM 2127) were obtained from Japan Collection of Microorganisms (Tsukuba, Ibaraki, Japan). The details of the strains used are available at https://www.jcm.riken.jp/cgi-bin/jcm/jcm_number?JCM=1233 and https://www.jcm.riken.jp/cgi-bin/jcm/jcm_number?JCM=2127. The strains were maintained at −80°C in tryptic soy broth (TSB, Merck, Darmstadt, Germany). They were recovered from a frozen stock and cultured on tryptic soy agar (TSA; Merck, Darmstadt, Germany) plates. Since the objective of this study was to investigate the response to desiccation stress of dried bacterial cells *per se*, considerable quantities of bacterial cells were needed for physical and microbiological analyses. To efficiently prepare and collect bacterial cells in quantity and exclude an effect of specific nutrition, we used tryptic soy broth (TSB) as a representative and standard culturing medium. The cultures were incubated at 37°C for 24 h, and an isolated colony of each bacterium was transferred to 5 ml of fresh TSB at 37°C for 24 h; thereafter, 100 μl of cultured C. sakazakii cells was added to 800 ml of TSB and incubated at 37°C for 48 h. The cultured cells were centrifuged (3,000 × *g*, 10 min), and the resulting pellets were washed with 30 ml of pure water. Then, the bacterial cell suspension with pure water was centrifuged once more with the same procedure as described above to obtain purified bacterial cell pellets.

The collected pellets of C. sakazakii cells were dried by one of two methods widely used for various kinds of foods in the food industry. One group of bacteria was subjected to air drying, which is based on the principle of diffusion. The bacterial cell pellets were placed in a biological safety hood at room temperature for 48 h to dry them completely. Another group of C. sakazakii cells was freeze-dried for 24 h in a freeze dryer (FDU-2200; EYELA, Tokyo, Japan) at −80°C and a pressure of <10 Pa. Both groups of dried samples were ground and powdered manually using a sterile mortar and pestle.

### Determination of the mechanical glass transition temperature (*T_g_*).

The powdered C. sakazakii cells were stored in airtight containers at four levels of relative humidity (% RH) and 4°C for 48 h to stabilize their a_w_ (3). The % RH was adjusted using a saturated aqueous salt solution (43% RH, potassium carbonate; 57% RH, sodium bromide; 75% RH, sodium chloride; and 87% RH, potassium chloride). The RH and temperature in the airtight container were checked continuously using a thermorecorder (TR-72wf; T and D, Nagano, Japan). The a_w_ of the bacteria was confirmed by a water activity meter (Aqualab 4TE; Decagon Devices, Pullman, WA, USA).

**FIG 5:**
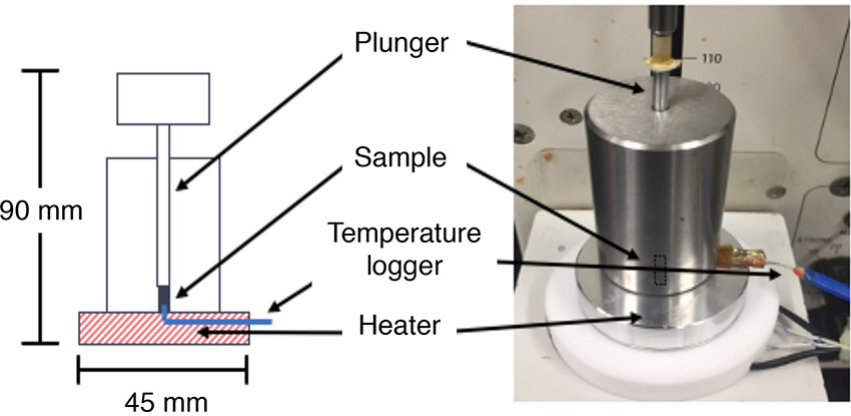
Schematic diagram explaining the thermal rheological analysis (TRA).

The *T_g_* of the a_w_-adjusted C. sakazakii cells was determined by thermal rheological analysis (TRA) as described in previous studies (37–40) and as shown in Fig. 5. A dried bacterial cell sample (ca. 100 mg) was placed in the forming die (φ = 3 mm) and compacted with a rheometer ca. 10 MPa. Subsequently, the sample was compressed at 7 MPa for 3 min and then heated at a rate of approximately 1.5°C/min until the temperature reached 120°C. Pressure-time data were collected with software attached to the rheometer. In parallel, a thermocouple was attached to the bottom of the forming die, and time-temperature data were collected every second using a datalogger. Accordingly, the relationship between pressure and temperature data during heating was determined. Since pressure reduction begins at the point at which the bottom temperature of the sample reaches the mechanical *T_g_*, the onset temperature of pressure reduction could be regarded as the *T_g_* of the sample. It is known that the onset *T_g_* determined by TRA is ∼5 to 10°C higher than that determined by differential scanning calorimetry (39).

### Survival kinetics of C. sakazakii cells under the selected temperature and relative humidity conditions.

Changes in the viable cell numbers of dried C. sakazakii cells stored in airtight containers maintained at different relative humidity levels (43% and 87%) at 4°C, 25°C, and 42°C were determined over time. The surviving cell numbers of C. sakazakii were determined periodically, after 1, 2, and 3 weeks of storage. Triplicate trials were conducted. Fifty milligrams of dried C. sakazakii cells was mixed with 500 μl of 0.1% peptone water, and then the bacterial cell suspension was serially diluted 10-fold. The diluted samples at each dilution level were plated onto duplicate TSA plates and incubated at 37°C for 24 h, and then the number of colonies was determined.

Triplicate samples were collected at each sampling time. The colony counts for the triplicate samples of each bacterium at each sampling interval were transformed to log CFU/g values, and the values for the triplicate samples were averaged to represent the number of viable cells at each sampling time.

The Weibull model, one of the most frequently used survival models (3, 6, 41), was used as a representative model. This model is described by the following formula:

(1) $$\log\;S\left(t\right)=\log\frac{N\left(t\right)}{N_{0}}=-b\times t^{n}$$

where *S*(*t*) is the momentary (“instantaneous”) survival ratio, *b* and *n* are parameters representing the survival rate and kinetics curve, respectively, and *N*(*t*) and *N*_0_ are the momentary and initial cell counts, respectively. The initial cell counts (*N*_0_) were assumed to represent the state of the samples immediately after drying by each drying method. The survival ratio under each temperature and RH condition was fitted using the Weibull function described by equation 1 via nonlinear least-squares regression.

### Statistical analysis.

Samples were run and analyzed in triplicate, and the results were averaged to represent the number of viable cells at each sampling time. The data are expressed as the mean values ± standard deviations (SD) and were analyzed using R statistical software (version 3.4.1 for Mac OS). One-way analysis of variance (ANOVA) was performed to compare the differences between different treatments. *T_g_* levels and survival ratios were compared using the Tukey-Kramer multiple-comparison test. Results with *P* values of <0.05 were considered statistically significant.

### Data availability.

All data generated or analyzed during this study are included in this article.
